# Fusion-based implementation of qLDPC codes with quantum emitters

**DOI:** 10.1038/s41534-026-01233-y

**Published:** 2026-04-13

**Authors:** Susan X. Chen, Matthias C. Löbl, Ming Lai Chan, Anders S. Sørensen, Stefano Paesani

**Affiliations:** 1 Quantum Engineering Centre for Doctoral Training, H. H. Wills Physics Laboratory and School of Electrical, Electronic, and Mechanical Engineering, University of Bristol, Bristol, UK; 2 NNF Quantum Computing Programme, Niels Bohr Institute, University of Copenhagen, Copenhagen Ø, Denmark; 3 Center for Hybrid Quantum Networks (Hy-Q), The Niels Bohr Institute, University of Copenhagen, Copenhagen Ø, Denmark; 4 Sparrow Quantum, Copenhagen Ø, Denmark

**Keywords:** Mathematics and computing, Optics and photonics, Physics

## Abstract

Quantum low-density parity check (qLDPC) codes offer higher encoding rate than topological codes, e.g. surface codes, making them favourable for practical, fault-tolerant quantum computing with low overhead. These codes are particularly well-suited for fusion-based photonic implementations as this platform readily supports non-local connections. We propose an architecture specifically tailored to quantum emitters which can implement any Calderbank-Shor-Steane (CSS) qLDPC code. In this architecture, the photonic resource states are deterministically produced via quantum emitters and a conditional repeat-until-success strategy is incorporated to achieve high photon loss tolerance. We simulate small exemplary Bivariate Bicycle qLDPC codes and analyse the performance of our constructions under relevant physical noise mechanisms, including erasures due to fusion failure or photon loss, as well as Pauli errors. We obtain performances comparable with topological architectures though with significantly higher encoding rates.

## Introduction

The development of reliable quantum computers critically depends on advancing fault-tolerant quantum computing architectures that provides access to large numbers of error-corrected qubits with practical quantum hardware. For the past decades, topological codes, such as the surface code, have been studied extensively from a theoretical perspective and have become the standard platform for developing these architectures. This is due to their local connections in a planar layout and their high error-correction thresholds^1–3^. These codes have also recently been experimentally demonstrated in fault-tolerant regimes^4^. Despite this, they suffer from very limited encoding rate, where each surface code patch is only able to encode a single logical qubit and the associated overhead to access large numbers of error corrected logical qubits pose a notable challenge for current and near-term quantum hardware.

In recent years, general (non-topological) classes of quantum low-density parity check (qLDPC) codes with significantly improved encoding rates and similar error correction performances have been developed^3,5^. However, this improvement comes with the challenge that very often long-distance connectivity is required when the qubits are arranged on a planar two-dimensional geometry^5,6^, posing difficulties for implementations in static solid-state qubit platforms, such as superconducting qubits and gate-defined quantum dots. Meanwhile, such long-range interactions can be implemented with other platforms. For example, photonic qubits can be transmitted over long distances using waveguides and optical fibres, and atomic qubit arrays are reconfigurable to enable interactions between distant sites^7,8^.

With such capabilities, these platforms can naturally benefit from the more efficient encoding of qLDPC codes, and architectures for atomic qubits have already been explored based on two-qubit gates between Rydberg atomic states^9^. Photonic qubits, however, lack direct qubit-qubit interactions and thus require significantly different constructions. One possibility encodes qubits into bosonic modes, enabling deterministic entangling interactions. Such an architecture with arbitrary qLDPC code input was recently proposed in ref. ^10^. However, it relies on GKP states which are experimentally challenging to generate and on operations tailored specifically to continuous-variable systems. An alternative approach uses single-qubit encodings, along with probabilistic two-qubit operations, so-called *fusions*^11,12^.

Fusion-based quantum computing (FBQC) has recently been developed as a method to build and analyse fault-tolerant constructions based on these gates, where operations are performed by consuming small entangled photonic resource states through fusions^13,14^. In this article we construct fusion-based logical memories based on qLDPC codes that offer better encoding rates, specifically Bivariate Bicycle codes^5^ and analyse their thresholds with phenomenological noise models as well as photon loss. This approach generalises to any Calderbank-Shor-Steane (CSS) qLDPC code and implements a logical memory solely through photonic fusions and resource states that can be deterministically generated via quantum emitters^15,16^.

## Results

### qLDPC codes

Quantum low-density parity check codes are stabiliser codes with low and constant qubit and check degrees. As a result, these codes are described by sparse parity check matrices^3^. Following standard notation, we present codes in terms of their parameters [[*n*, *k*, *d*]], where *n* is the number of physical qubits, *k* is the number of encoded logical qubits, and *d* is its distance, i.e. the smallest possible Hamming weight of a logical operator. We focus on qLDPC codes that are CSS where checks are made up of solely Pauli *X*- or *Z*-operators and therefore allow the two types of error to be detected and corrected independently.

Recently, qLDPC code families with encoding rates *k*/*n* greater than those of topological codes have been studied^17,18^. It has also been shown that there exist codes with *asymptotically good* parameters^19,20^, i.e. codes for which the number of encoded qubits and the distance both scale linearly with the number of physical qubits. Such codes offer a clear route to reducing overhead in fault-tolerant schemes.

We consider the recently proposed Bivariate Bicycle qLDPC code family^5^ (See Supplementary Information III). These are the first known examples of codes that can compete with the surface code in terms of logical error rate while offering a higher encoding rate. The choice to focus on Bivariate Bicycle codes is arbitrary, and serve solely as an example study. Our approach is applicable to any qLDPC CSS code and could therefore also be applied to a larger class of codes with proven asymptotically constant encoding rate^19,20^.

### Construction

Measurement-based quantum computing models temporally evolve the encoded logical information by performing single-qubit measurements on a large entangled graph state. A graph state, $| G\rangle$, is represented by an undirected graph *G* = (*V*, *E*) where the vertices *V* are qubits initialised in the state $| +\rangle =\frac{1}{\sqrt{2}}(| 0\rangle +| 1\rangle )$ and edges *E* are controlled-phase operations ${U}_{c}^{a,b}$ between the vertices *a*, *b* that they connect:

1 $$| G\rangle =\mathop{\prod }\limits_{a,b\in E}{U}_{c}^{a,b}{| +\rangle }^{\otimes V}$$

Graph states are +1 eigenstates of their stabiliser generators

2 $${K}_{i}={X}_{i}\mathop{\prod }\limits_{j\in {N}_{i}}{Z}_{j},$$

where *N*_*i*_ is the neighbourhood of qubit *i*^21^, and *X*_*i*_ and *Z*_*j*_ are Pauli operators.

**Fig. 1:**
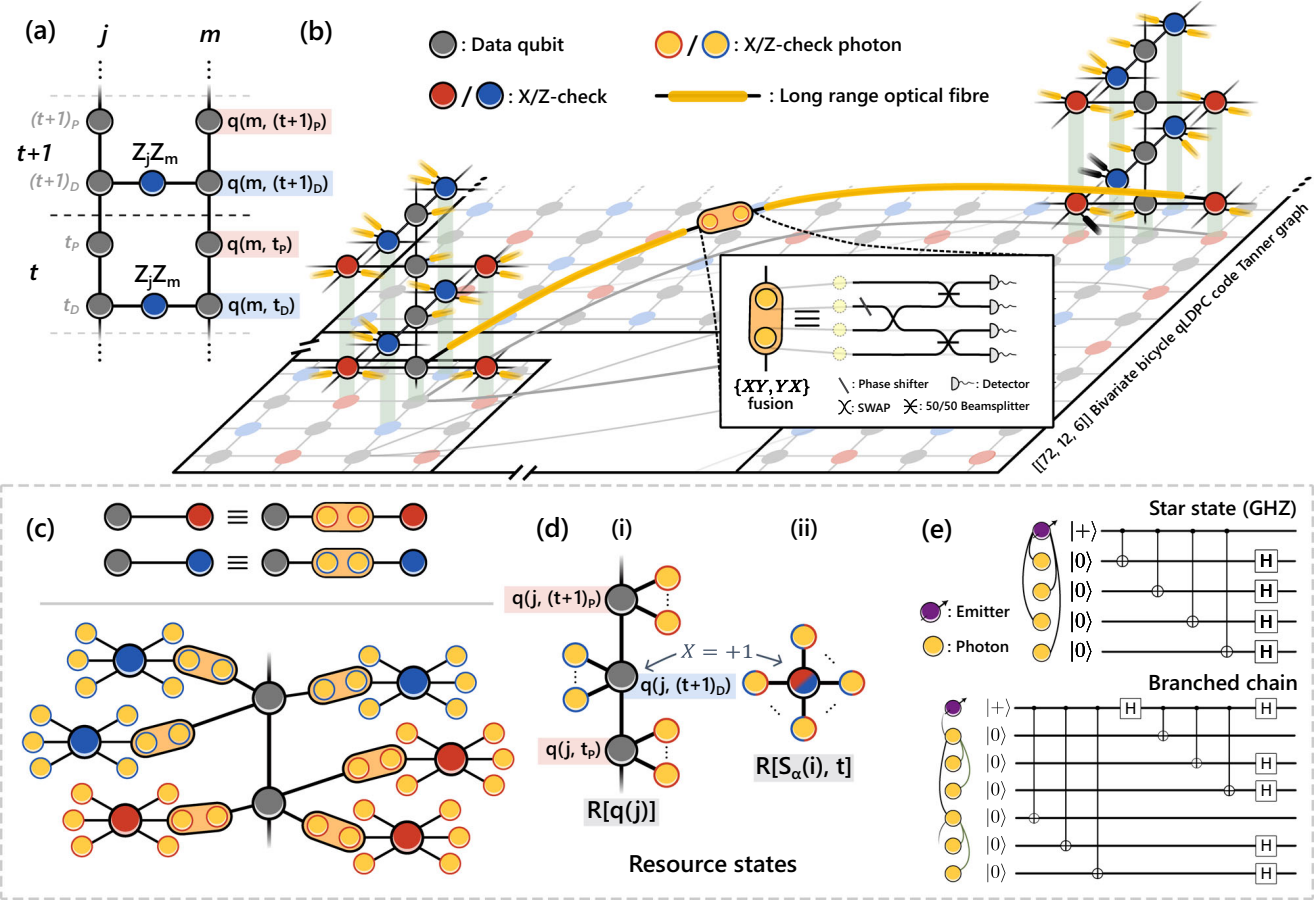
Outline of the architecture. **a** Setup for the measurement of parity check *Z*_*j*_*Z*_*m*_ of two foliated data qubits *j* and *m* at time intervals *t* and *t* + 1 by coupling their $t{{\prime} }_{D}$ layer qubits to ancillary qubits (blue). In between the *Z*-parity checks, parity check of the *X*-operators are performed with other data qubits. **b** Foliated cluster state lattice of the [[72, 12, 6]] Bivariate Bicycle qLDPC code (partially shown in a Toric layout) from ref. ^5^, where each *layer* constitutes only *X* or *Z*-checks upon measuring the qubits. In addition to the four nearest neighbour links, each qubit also has two long-range connections, such as the ones shown for a sub-set of nodes in the Tanner graph. In our fusion-based decomposition of the lattice, each data qubit to ancilla qubit link is split and realised with an 〈*XY*, *YX*〉 photonic fusion for which a setup is shown in dual-rail encoding. **c** An illustration of how a data qubit completes its one error detecting round (layer) of checks in the lattice via fusions. **d** The two types of resource states that make up the fusion lattice. (i) Data branched chains: Linear cluster states of length 2*T* with *η*_*Q*_/2 leaves per layer. (ii) Ancilla GHZ states: Shown as (*η*_*A*_ + 1)-star graph states for clarity. We note that all qubits which are not explicitly drawn as fusion photons (yellow) are projected onto the measurement outcome *X* = + 1. **e** Deterministic generation protocols for the star and branched chain photonic graph states using quantum emitters as detailed in refs. ^16,31^.

Typically, fault-tolerant circuit-based implementations of a CSS (Foliation can also be applied to non-CSS codes^22^, though in the rest of this manuscript we will assume CSS constructions.) code require *d* repetitions of its stabiliser measurements. This temporal sequence of operations can be replicated in a measurement-based sense by *foliating* the underlying *base* error-correcting code^23,24^. Foliation transforms the code into a graph state with a layered structure, replacing time with an extra spatial dimension perpendicular to the layers. (We will also refer to this graph state as a cluster state or lattice in the following). Using the cluster state, one can perform all repeated check measurements by measuring the state layer by layer^22^. As illustrated in Fig. 1a, this graph state has alternating layers of bipartite graphs where data qubits would be connected to ancilla qubits according to the structure of the Tanner graph associated with the *Z*- and *X*-type parity checks of the underlying CSS code. The data qubits are connected between these layers^23^. This structure encodes data qubits of the base code in a one-dimensional chain of length 2*T* qubits, where we label the *j*^*th*^ such data qubit as *q*(*j*) and *T* is the number of stabiliser measurement rounds of the logical memory. The logical information of the chain is locally accessible and can be propagated along the chain, analogously to temporal evolution, by performing single-qubit *X*-measurements on *past qubits* of the chain. Figure 1a shows an example of the repeated measurement of a small parity check between two foliated data qubits, *j* and *m*. At each time-step layer, stabilisers are measured by measuring their ancilla qubits in the *X*-basis. Due to the way chain logical operators are accessed, the *Z* and *X* stabilisers are measured sequentially, so two ‘temporal’ layers *t* = {*t*_*D*_, *t*_*P*_} are required to complete one full measurement round of the code. Throughout the rest of the paper, we will refer to the layer in which only *Z*-stabilisers are measured as the dual layer, *t*_*D*_, and the layer in which *X*-stabilisers are measured as the primal layer, *t*_*P*_. The physical qubits on the chain from the *j*^*th*^ base data qubit are labelled *q*(*j*, *t*_*D*_) and *q*(*j*, *t*_*P*_).

The cluster state resulting from the procedure described above possesses graph state stabilisers by definition. By multiplying graph state stabiliser generators, one finds that there exists two sets of regularly structured local detectors spanning three layers of the foliated lattice^23^. These detectors form *unit cells* of two separate lattices: *X*-type and *Z*-type (generalisations of primal and dual lattices in topological code foliations).

The *X*-type detectors detect changes in parity of the *X*-checks of the base error-correcting code labelled *S*_*X*_(*i*), and thus errors which may contribute to a logical *X* error in the foliated code. We denote ${{\mathcal{X}}}_{i,t}$ as the set of qubits on which there is Pauli-X operator support for the detector associated with the *i*^*th*^*X*-check at time interval *t*:

3 $$\begin{array}{rcl}{{\mathcal{X}}}_{i,t} & := & \mathop{\underbrace{\{{s}_{X}(i,{t}_{P})\}}}\limits_{1\mathrm{st}\,\mathrm{layer}}\cup \mathop{\underbrace{\{q(j,{(t+1)}_{D})| j\in {S}_{X}(i)\}}}\limits_{2\mathrm{nd}\,\mathrm{layer}}\\ & & \cup \mathop{\underbrace{\{{s}_{X}(i,{(t+1)}_{P})\}}}\limits_{3\mathrm{rd}\,\mathrm{layer}}.\end{array}$$

Here, we label *s*_*X*_(*i*, *t*_*P*_) as the physical ancilla qubit associated with the *i*^*th*^*X*-check *S*_*X*_(*i*) of the base code within layer *t*_*P*_ of the foliated lattice, and *q*(*j*, (*t*+1)_*D*_) as the physical data qubit in the dual layer at the time interval *t* + 1 corresponding to the *j*^*th*^ foliated chain. A logical *X* operator of the cluster state, or *correlation surface*, is effectively a surface that traverses the foliated length 2*T* of the lattice. This surface has weight on chain qubits associated with the corresponding, say *k*^*th*^, logical *X* operator of the base code ${\overline{X}}_{k}^{base}$ across all *t*_*D*_ layers. In other words, the *k*^*th*^ logical *X*-operator ${\overline{X}}_{k}$ for the lattice is

4 $${\overline{X}}_{k}=\mathop{\prod }\limits_{{t}_{D}=1}^{T}{\overline{X}}_{k}^{base}({t}_{D}).$$

Analogously, a *Z*-type detector cell helps detect physical errors which may contribute to logical *Z* errors and has a comparable structure, spanning the layers {*t*_*D*_, *t*_*P*_, (*t*+1)_*D*_}:

5 $$\begin{array}{rcl}{{\mathcal{Z}}}_{i,t} & := & \mathop{\underbrace{\{{s}_{Z}(i,{t}_{D})\}}}\limits_{1\mathrm{st}\,\mathrm{layer}}\cup \mathop{\underbrace{\{q(j,{t}_{P})| j\in {S}_{Z}(i)\}}}\limits_{2\mathrm{nd}\,\mathrm{layer}}\\ & & \cup \mathop{\underbrace{\{{s}_{Z}(i,{(t+1)}_{D})\}}}\limits_{3\mathrm{rd}\,\mathrm{layer}}.\end{array}$$

The *k*^*th*^ logical *Z*-operator ${\overline{Z}}_{k}$ is similarly defined

6 $${\overline{Z}}_{k}=\mathop{\prod }\limits_{{t}_{P}=1}^{T}{\overline{Z}}_{k}^{base}({t}_{P}).$$

Using the method in ref. ^23^, we may foliate any CSS qLDPC code with parity check matrices *H*_*X*_ and *H*_*Z*_. Here, we consider codes that, in addition, have constant qubit-degree *η*_*Q*_ and constant check-degree *η*_*A*_. This is not required for our approach to apply but simplifies the fusion-based construction (Otherwise, variations of the two types of resource states (see below) are required). The qubit-degree is the number of checks each data qubit is connected to, and check-degree is the number of data qubits involved in each check. For Bivariate Bicycle codes, *η*_*Q*_ = *η*_*A*_ = 6 with every qubit being involved in three *X-* and three *Z*-type checks. In our analysis of the codes, we choose the number of measurement rounds, *T*, to be the error distance *d* of the underlying code.

To reduce the demands of producing the lattice as a large entangled cluster state, we consider a fusion-based approach where the foliated system is decomposed into smaller resource states and type-II fusion operations^11^ between them (shown in Fig. 1b for the [[72, 12, 6]] Bivariate Bicycle qLDPC code). Fusions are probabilistic Bell state measurements on two qubits *A*, *B* that measure joint parities such as 〈*X*_*A*_*X*_*B*_, *Z*_*A*_*Z*_*B*_〉 upon success^12,25,26^ (other parity measurements can be realised via rotation with single-qubit gates^25^—see Supplementary Information II for details). Naively, these operations succeed with a 50% probability in photonics^11^, however, this probability can be boosted with the addition of ancillary photon pairs as part of the fusion gate^27–29^.

In the considered foliated structure, the data qubits are connected between different layers, forming chain structures. Within every layer, the graph is bipartite as data qubits are only connected to ancilla qubits (see Fig. 1c). This structure gives rise to a very natural fusion-based construction using branched chain graph states (caterpillar trees in graph theory^30^) which are linear cluster states with additional *leaf* qubits connected to qubits of the linear chain, and star-shaped graph states. The data qubits form the central path of branched chains (grey in Fig. 1c, d), and ancilla qubits form the central qubits of star-shaped graph states (red, blue in Fig. 1c, d) where they are connected to the branched data qubit chains by fusions (yellow in Fig. 1c, d). Importantly, branched chains constitute exactly the class of states that the scheme from ref. ^31^ can generate using a single quantum emitter (Furthermore, by applying Hadamard gates on the leave qubits of a branched chain it is converted into a redundantly encoded linear cluster state^15,16^ which enables using encoded rather than physical fusions^32^). This approach employs a sequence of spin gates on a quantum emitter and optical excitation which induces photon-emission from the emitter^33^. Figure 1e shows the quantum circuit that deterministically generates a branched chain and a star-shaped graph state, which is a branched chain of length one and is locally equivalent to the GHZ. Below we give a more detailed description of the involved resource states (1) data branched chains, and (2) ancilla GHZ states.

*Data branched chains* are branched chains of length 2*T* with *η*_*Q*_/2 branches stemming from each physical qubit of the chain. Being our equivalent of a *foliated qubit*, the lattice contains one data branched chain per underlying code qubit, hence we label these resource states *R*[*q*(*j*)] where *q*(*j*) is the *j*^*th*^ base code data qubit. The branched qubits of *R*[*q*(*j*)] at layer *t*_*D*_(*t*_*P*_) will go into fusions which complete all the *Z*(*X*) code checks that the code data qubit *q*(*j*) takes part in. All grey qubits in Fig. 1d(i) are virtual qubits assumed to be measured in the *X*-basis with the measurement outcome +1 as discussed below.

*Ancilla GHZ states* are visually represented, in Fig. 1d(ii), as star graphs with *η*_*A*_ leaves with the central qubit measured and projected into +1 in the *X*-basis. It can be shown that the resulting state from this requirement is a GHZ state on *η*_*A*_ qubits. The lattice comprises one ancilla-GHZ state per code check involved in each layer which we label *R*[*S*_*α*_(*i*), *t*], where *S*_*α*_(*i*) denotes the *i*^*th*^ base code check of type *α* ∈ {*X*, *Z*} as above and *t* is the time interval at which the check takes place. These ancilla states fuse with the leaf qubits of the data resource states that are part of the check (Fig. 1c—for details of choice of fusion type see Supplementary Information II).

We remark that the central qubits of the branched chain and (*η*_*A*_ + 1)-star graph are virtual^13,34^: they are used to simplify the visualisation of the graph, but in practice the physical state to be generated corresponds to the graph obtained measuring the virtual qubits in *X* and with outcome +1 (see Supplementary Information I for a depiction of the reduced resource states and resulting lattice). We note that the state after applying a Pauli measurement on a branched chain is local Clifford equivalent to another branched chain (This is because a Pauli measurement creates a vertex minor^35^ of the graph and a vertex minor cannot have an increased linear rank width.). Using the scheme from ref. ^31^, one thus can directly generate the state resulting from measuring the central qubits of the branched chain or (*η*_*A*_ + 1)-star graph in the *X*-basis. In other words, these virtual qubits do not need to be generated in the first place and fusions are the only measurements that need to be applied, giving rise to an entirely fusion-based scheme. Since photons that are not generated cannot be lost, this scheme will have a higher erasure threshold.

When quantum emitters are employed, it turns out that the above fusion-based scheme does not represent the optimum strategy to reduce the effect of photon loss. More practical is likely to be a scheme where not all fusion photons are generated at once but rather on demand and fusions are repeated until success (see Supplementary information I).

### Threshold analysis

Using the method detailed above, we construct lattices of varying sizes of Bivariate Bicycle qLDPC codes. We test the lattices’ tolerance to both error and erasure of fusion outcomes. An independent and identically distributed (i.i.d.) fusion noise model is used^13^, where logical error probabilities are determined based on a physical noise rate *p* occurring independently for each fusion based on an error probability *p*_*e*_ and an erasure probability *p*_*l*_. For decoding, we use a modified version of the union-find decoder from ref. ^36^ since it works for non-topological qLDPC codes and can handle Pauli errors and erasures simultaneously (see ‘Decoder details’ section under Methods for more details).

Topological qLDPC codes, like the surface code, have a well-defined error threshold: for error rates below the error threshold, an arbitrary low logical error rate can be obtained by increasing the code size. For general qLDPC codes, however, there is no systematic way of scaling the size of the code. Therefore, we evaluate the fault-tolerant performance of the qLDPC lattices with respect to their so-called *pseudo-thresholds*. A pseudo-threshold represents the physical error rate at which the probability for an error on one out of *k* logical qubits (the logical error rate $\overline{p}$) equals ${p}_{k}^{phys}=1-{(1-p)}^{k}$, the probability that at least one out of *k* physical qubits has an error - this we refer to as the *break-even equation*. Below the pseudo-threshold the logical error is smaller than the physical, ${p}_{k}^{phys} > \overline{p}$, making it beneficial to use encoded rather than physical qubits. In general, the noise is a combination of fusion error and erasure. We consider a break-even equation where *p* = (1 − *p*_*l*_)*p*_*e*_ + *p*_*l*_/2 (See Section ‘Details of numerical simulations’ for details). This represents the probability *p* of an error occurring, either without erasure or due to an erasure, where erasures introduce errors with a probability of 50%^37^.

**Fig. 2:**
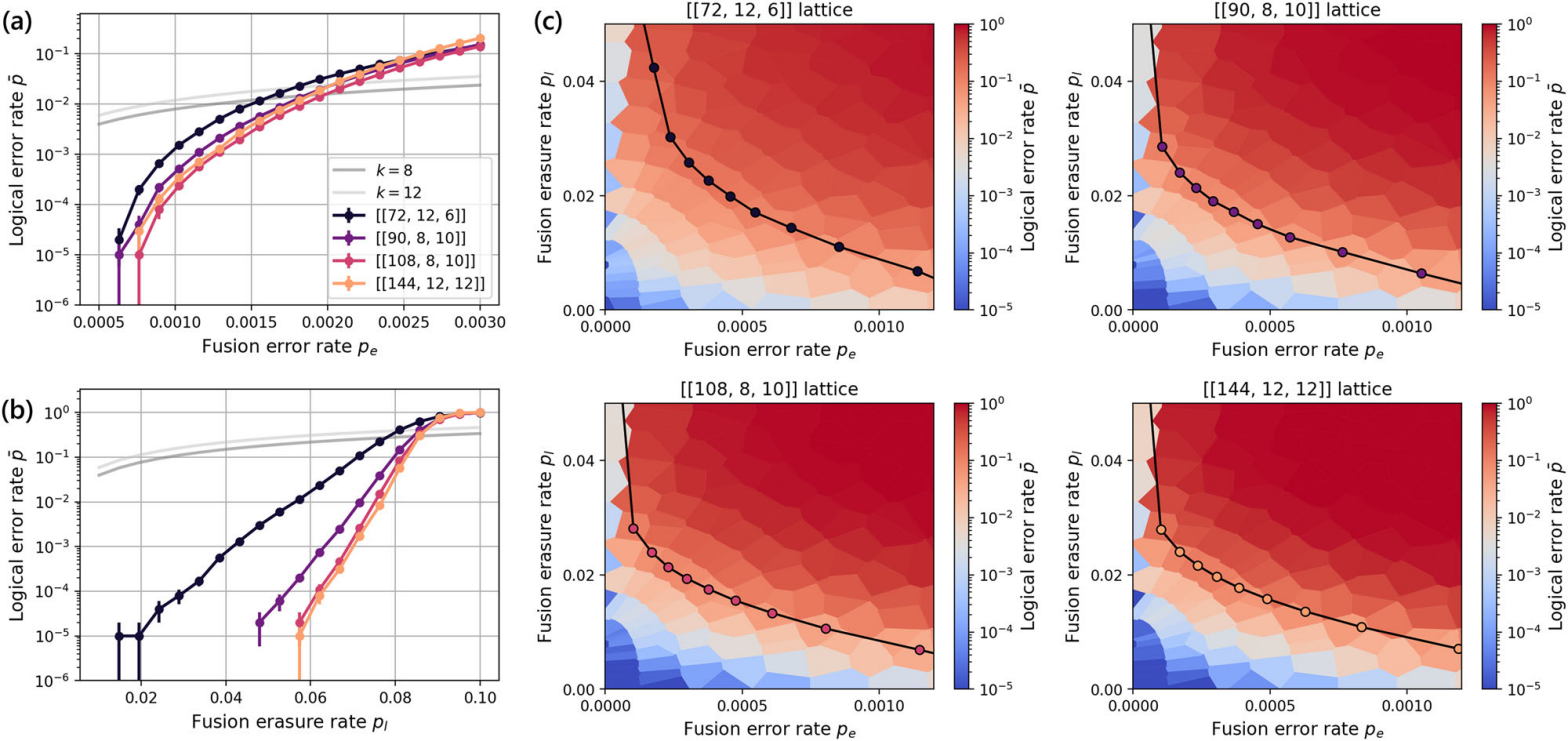
Logical error rates $\overline{p}$ for lattices constructed from small examples of Bivariate Bicycle qLDPC codes. Given **a** fusion error only and **b** fusion erasure only. The grey curves labelled *k* = 8 and *k* = 12 represent the probability of having at least one error across the *k* physical qubits at a given physical noise rate, i.e. ${p}_{k}^{phys}=1-{(1-p)}^{k}$. The pseudo-threshold is defined as the point where the logical error rate of the lattice intersects with its corresponding grey curve, a.k.a., $\overline{p}={p}_{k}^{phys}$. Lower bounds on the truncated error bars in (**a**, **b**), not shown for ease of visualisation, are 5 × 10^−11^. **c** Logical error rate as a function of fusion erasure *p*_*l*_ and error *p*_*e*_ rates for each lattice. The points on each colour plot correspond to the said lattice’s pseudo-threshold surface, such that the area below the curve can be interpreted as a *pseudo-correctable region*.

Pseudo-thresholds are found by performing Monte Carlo simulations. We present the logical error rates for Bivariate Bicycle code lattices in Fig. 2a, b, under independent sweeps of phenomenological fusion error and erasure probabilities, and plot the break-even equations for different number of encoded qubits *k* = 8, 12 as grey curves. Each logical error rate point is the mean over 10^5^ independent simulation samples, and the error bars shown represent ±1 standard deviation considering Poissonian noise due to the finite number of samples. The colour plots in Fig. 2c show logical error rates for each lattice sweeping through error and erasure noise combinations in the grid (while also including the additional data from pseudo-threshold analysis below) where the points mark a pseudo-threshold curve for the corresponding lattice. In order to obtain these points, we introduce a parameter *x*, which we call the *noise scaling factor*, that linearly parameterises a line at equally distributed set of angles from the origin. At each angle, we recover logical error rates and pseudo-thresholds by sweeping over *x* and calculating the intersection with the modified break-even equation discussed above. As expected, it can be seen from Fig. 2a, b that as the code size increases, the pseudo-thresholds for fusion error and fusion erasure both increase, with the [[144, 12, 12]] code having an error pseudo-threshold of 0.2% and an erasure pseudo-threshold of 9%. We note that pseudo-thresholds should not be taken as a definitive performance metric. In particular, while the [[72, 12, 6]] qubit code has an unusually high comparative pseudo-threshold, its logical error suppression at low physical error rates is worse despite this.

### Repeat-until-success (RUS) fusions

**Fig. 3:**
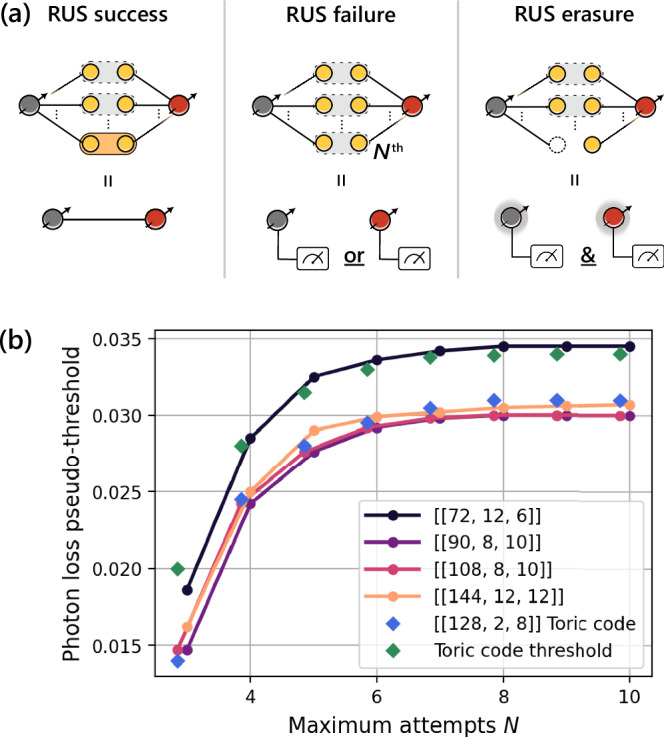
Repeat-until-success strategy and boosted photon loss pseudo-thresholds. **a** Modified repeat-until-success strategy where RUS success, failure and erasure result in no action, measurement of either spin, or measurement of both spins, respectively. Whether neighbouring RUS fusions are performed is adaptively decided based on the RUS fusion outcome (see Supplementary Information I). **b** Photon loss pseudo-threshold as a function of the maximum number *N* of repetitions in a RUS fusion, shown for small Bivariate Bicycle qLDPC code lattices. The blue points show the pseudo-threshold for [[128, 2, 8]], a comparable instance of the Toric code lattice and the green points show the actual threshold for Toric code lattices.

Above, we have used abstract erasure *p*_*l*_ and error *p*_*e*_ rates, referring to the results of the fusions. A different question is how these quantities relate to the underlying physical parameters. To investigate this, we evaluate the tolerance of physical lattice constructions to photon loss, as it is the dominant source of noise in photonic platforms. The phenomenological erasure thresholds obtained above are too low for an implementation with standard linear optics Bell-state measurements^11^ that fail with 50% probability. To overcome this issue, it is thus necessary to boost the fusion probability. To achieve boosted fusion, a particularly promising technique for quantum emitters is a repeat-until-success (RUS) scheme, repeating physical fusions until success^32,38,39^. At each layer, the quantum emitters stay part of the resource states and emit photons that undergo fusion measurements until the fusion is successful, lost, or a maximum number of attempts *N* is reached and all of the physical fusions have resulted in failure (see Fig. 3a). We note that fusions only occur within independent layers of the foliated structure. This enables applying the emitter-based repeat-until-success fusions with the capability for synchronous scheduling^40^. RUS fusion outcomes can be sampled based on the standard analytical model described in ref. ^38^ where all fusions within a given layer are done in parallel, which we refer to as *standard RUS*. We consider a *modification* of standard RUS such that the fusions are instead performed sequentially. Note that also strategies in between are conceivable, where RUS fusions are grouped and RUS fusions of the same group are executed in parallel while the groups are executed sequentially.

The two above strategies can be understood in the picture of cluster states and the effects of RUS erasures and failures on bonds in the built cluster state lattice. *RUS erasure* causes two detector cells in both the *X*-type and *Z*-type syndrome lattices to merge into a higher-weight *supercell*^41^. Concretely, the detector cells are merged that share the bond that the successful RUS fusion would generate between two spins. In practice, both spins are measured in the *Z*-basis (see Fig. 3a RUS erasure). This is necessary because is unclear if the fusion photon connected to the left or right spin was lost, and losing one fusion photon has thus the same effect as losing both fusion photons. *RUS failures* equate to missing bonds and are less detrimental because only a merge in one of the two lattices is required, and only one of the spins associated with the failed fusion must be measured in the *Z*-basis^42^ (see Fig. 3a RUS failure). While standard RUS will always measure out the spin associated with the ancilla qubit, in the modified strategy, we randomise the choice of measuring either end of the fusion to help balance cell merges across both syndrome lattices^42^. In addition, by performing fusions sequentially instead of simultaneously, we use the outcomes to decide about the necessity of subsequent neighbouring fusions. To increase loss tolerance, unnecessary future fusions are omitted, in particular, a fusion may be skipped if either of the spins involved needs to be measured in the *Z*-basis because of a missing bond (due to a previously failed RUS fusion). We note that a more detailed explanation of both the standard and modified variants of RUS can be found in Supplementary Information I.

With fusion outcomes sampled based on our modified RUS strategy, we perform Monte Carlo simulations on 10^5^ trials for fusion lattices built from various sizes of Toric and Bivariate Bicycle qLDPC codes. We present the photon loss pseudo-thresholds for Bivariate Bicycle code lattices in Fig. 3b. Similar to the previous section, we compute the pseudo-thresholds as the photon loss probabilities, which incur errors of at least one of the *k* logical qubits with the same probability as for the physical qubits. For the [[144, 12, 12]] code lattice, the pseudo-threshold saturates around 3% at *N* = 8. Such photon loss values may be reached with future developments of photonic platforms^7^. In Fig. 3b, we compare the results to the pseudo-threshold for photon loss of the [[128, 2, 8]] instance of the Toric code. This is the Toric code instance closest in qubit count to the other codes, enabling a direct comparison. The photon loss pseudo-threshold for [[128, 2, 8]] is seen to almost entirely overlap with those of the qLDPC codes (with the exception of the [[72, 12, 6]] code). Considering that the latter allow for much larger numbers of encoded qubits, this makes them highly attractive provided that experiments can be advanced to reach the thresholds.

We also show the actual threshold for Toric code lattices (in green), not to be confused with pseudo-thresholds at fixed code sizes, where it levels off to a maximum at 3.4% for *N* = 8. Due to our modified RUS procedure this is higher than the RUS loss threshold quoted in ref. ^38^ of 2.75% for an equivalent implementation of the Toric code.

## Discussion

We have presented an explicit method to construct cluster state lattices for FBQC from arbitrary CSS qLDPC codes. For a given input error-correcting code, the resulting lattice implements a logical memory by performing projective two-qubit fusion measurements between two distinct types of resource states. Although FBQC schemes are platform-agnostic^13^, we focus on photonics where fusion operations between qubits originating from different parts of the lattice can easily be implemented by routing photons to detectors through optical fibres. The two families of photonic resource states required in this particular decomposition are readily accessible as they have been experimentally generated through deterministic spin-optical excitation protocols^43–46^.

Furthermore, we employ a conditioned repeat-until-success strategy to boost photon-loss thresholds. This upgrades physical fusions to encoded fusions and uses local information from previous RUS outcomes to inform whether to perform subsequent surrounding fusions. This adaptive strategy avoids unnecessary fusions and thus reduces photon loss. Note that other threshold boosting strategies, such as dynamic biasing of failure bases based on global information have also been proposed in the literature^47^. Combining these strategies with our approach is an interesting next step to improving the loss tolerance of such constructions.

In general, qLDPC codes are highly attractive as they allow much more efficient encoding of information with dramatically reduced overhead in resources. The fact that this only comes with a modest increase in (pseudo-)threshold compared to the Toric code is a major asset of the considered approach. So far we have only considered a small subset of qLDPC codes. Since qLDPC codes are much less studied than, e.g. topological codes, it is likely that one can find codes that are more efficient than the ones considered here. It would be highly interesting to explore the full space of other qLDPC codes which offer better encoding rates and distances. Potentially, this could bring the threshold even closer to the Toric codes or reduce the resource overhead. Finally, with a particular physical platform in mind, incorporating more realistic noise models, e.g. spin errors, into simulations such as those in ref. ^40^ would provide important guidelines for future hardware developments towards the goal of realising fault-tolerant photonic quantum computers.

## Methods

### Details of numerical simulations

The pseudo-threshold points (Fig. 2) were found by simulating fusion lattices of size *T* = *d* with periodic boundary conditions under different noise settings. We consider only the *X*-type lattice in these simulations due to the *Z*-type being equivalent (the parity check matrix *H*_*X*/*Z*_ equivalence for all Bivariate Bicycle qLDPC codes implies that the distance for *X* and *Z* errors is the same^5^).

**Fig. 4:**
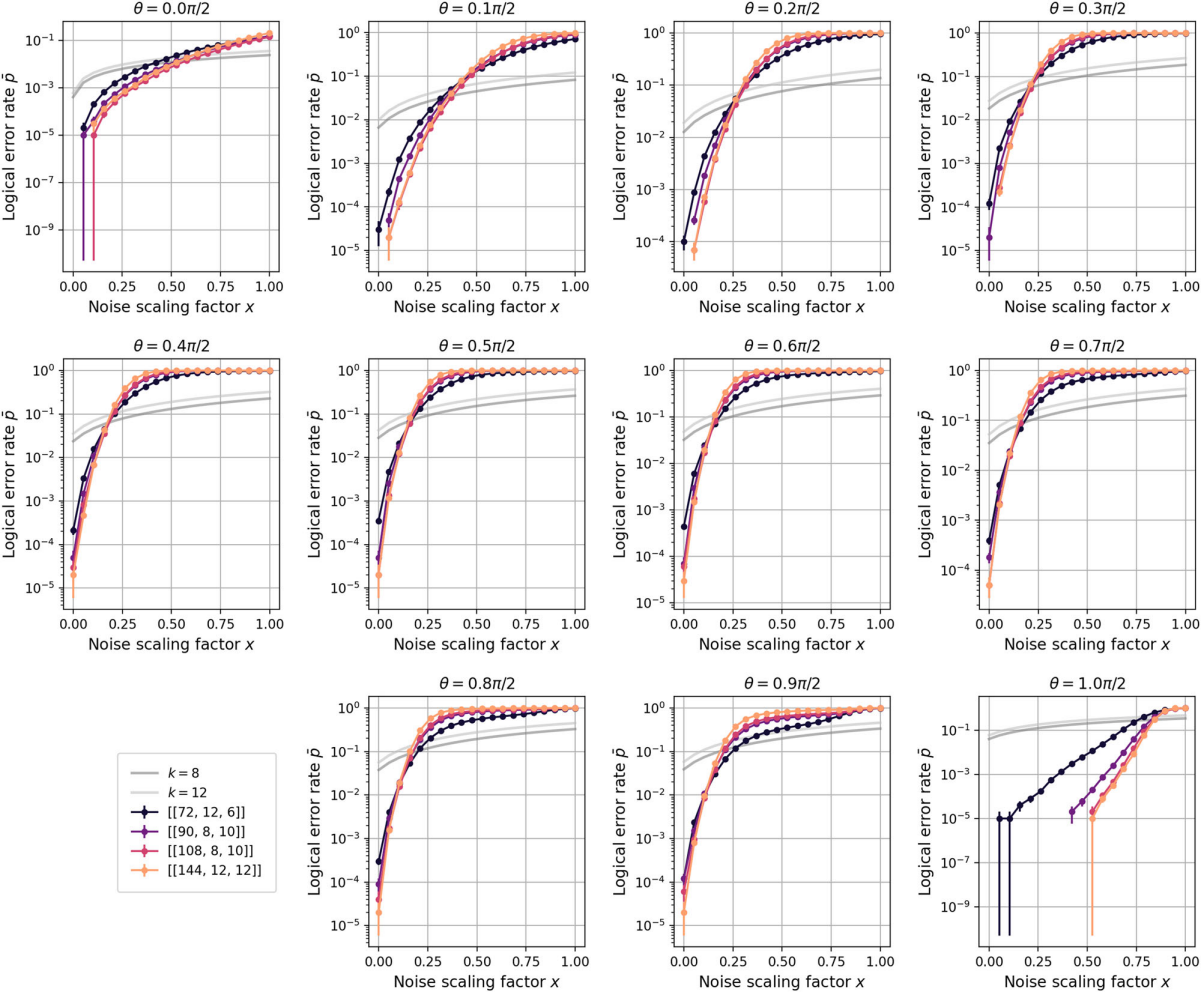
Logical error rates $\overline{p}$ against linearised noise parameter *x* for lattices constructed from small examples of Bivariate Bicycle qLDPC codes. Here $({p}_{e},{p}_{l})\propto (x\widetilde{{p}_{e}}\cos \theta ,x\widetilde{{p}_{l}}\sin \theta )$ up to additive offsets determined by minimum probabilities ${p}_{e}^{min}$ and ${p}_{l}^{min}$. The grey curves are the break-even equations for *k* = 8, 12 where its intersection with the data is the pseudo-threshold. The figure shows the series of noise combination sweeps of the error/erasure phase space given by angle *θ*, where *θ* = 0 is the error-only case and *θ* = *π*/2 is the erasure-only case.

We analyse performances by varying the fusion error *p*_*e*_ and erasure rates *p*_*l*_, which together define a two-dimensional phase space. This combined noise space is bounded by ${p}_{e}\in \{{p}_{e}^{\min },{p}_{e}^{\max }\}=\{5\cdot 1{0}^{-4},3\cdot 1{0}^{-3}\}$ and ${p}_{l}\in \{{p}_{l}^{\min },{p}_{l}^{\max }\}=\{1{0}^{-2},1{0}^{-1}\}$, as used to produce Figs. 2 and 4. We sweep across it along lines of constant angle *θ* where *θ* is taken from a set of equally distributed angles from the origin of the phase space parameterised by polar coordinates. Each sweep line is linearly parameterised by a variable *x*, the *noise scaling factor*, such that the corresponding mapping between fusion noise and *x* is given by

7 $$\begin{array}{rcl}{p}_{e} & = & (x\widetilde{{p}}_{e}+{p}_{e}^{min})\cos \theta \\ {p}_{l} & = & (x\widetilde{{p}}_{l}+{p}_{l}^{min})\sin \theta ,\end{array}$$

where $\widetilde{{p}}_{e}={p}_{e}^{max}-{p}_{e}^{min}$ and $\widetilde{{p}}_{l}={p}_{l}^{max}-{p}_{l}^{min}$. Here *θ* = 0, *π*/2 are lines of error and erasure only. With an i.i.d. (independent and identically distributed) model of both error and erasure of the fusions, we run 10^5^ Monte Carlo simulations with the modified union-find decoder (see ‘Decoder details’ section under Methods for details). Data from the complete simulation result for all noise combination sweeps is shown in Fig. 4, where the error bars shown represent ±1 standard deviation about the mean considering Poissonian noise due to the finite number of samples.

Erasure occurs with probability *p*_*l*_ and corresponds to a measurement with unknown outcome. Thus, we can assume that the corresponding qubit has an error with probability 0.5^48^. When there is no erasure (probability 1 − *p*_*l*_), an error occurs with the Pauli error rate *p*_*e*_. The overall error rate is therefore:

8 $$p=\frac{{p}_{l}}{2}+(1-{p}_{l}){p}_{e}.$$

We define the concept of a pseudo-threshold as the physical noise rate *p* when the probability that at least one of the *k* logical qubits has an error $\overline{p}$ is equal to the probability that one of the *k* physical qubits has an error ${p}_{k}^{phys}=1-{(1-p)}^{k}$. We refer to this as the *break-even equation* and it is plotted as grey curves in Figs. 2 and 4.

To produce Fig. 2c, we sweep an evenly distributed grid of points in the noise phase-space and similarly run the decoder on 10^5^ samples of each point. From this simulation, we construct a two-dimensional colour plot showing $\overline{p}$ as a function of *p*_*e*_, *p*_*l*_ where the Voronoi cell around every grid point is assigned its value of $\overline{p}$.

### Decoder details

To determine threshold error and loss rates of the foilated qLDPC codes, we need a decoder that can handle losses/erasures and also works for non-topological qLDPC codes. We chose a modified version of the union-find decoder^37^, which was presented in ref. ^36^.

The original union-find decoder^37^ works in two steps: first, it grows clusters on the Tanner graph starting from erasures and non-zero syndromes until all clusters are valid, where valid means that a cluster is decodable by only qubits within the cluster. For topological codes this so-called *syndrome validation* is easy as a cluster is valid/decodable if and only if it has an even parity of non-zero syndromes^37^. Secondly, clusters are decoded, which for topological codes, works with the peeling-forest decoder from ref. ^48^.

The studied qLDPC codes^5^ are non-topological and, therefore, neither syndrome validation by checking the cluster parity nor the peeling forest decoder can be applied. For this reason, we employ a modified union-find decoder^36^ that performs syndrome validation and decoding by solving the equation *σ* = *H*_*r*_ ⋅ *e* via Gaussian elimination. Here *σ* is an ${{\mathbb{F}}}_{2}$ vector of the cluster’s syndromes, *e* is an ${{\mathbb{F}}}_{2}$ vector with the cluster’s data qubits and *H*_*r*_ is the parity check matrix corresponding exclusively to this cluster. When a solution is found, it directly provides a decoding for the cluster. If there is no solution, the cluster cannot be decoded and is grown further. The cluster growth is performed node-by-node following breadth-first Tanner graph traversal, starting from erased qubits and non-zero syndromes. Cluster growth starting from erased qubits is performed first. Afterwards, we sort the nodes listed for subsequent cluster growth such that those belonging to invalid clusters are listed first. This sorting is the only difference to Algorithm 3 from ref. ^36^ and improves the logical error rates in the regime of high erasure rates. Finally, the subsequent cluster growth is performed in double steps on the Tanner graph such that cluster boundaries are syndrome checks and not data qubits^36^. This makes sure that decoding a cluster does not flip syndromes that are not part of it.

## Supplementary information

Supplementary Information

## Data Availability

The code and data generated for this article are openly available at the Github repository https://github.com/susanxschen/qldpc-fusion-lattices.

## Code availability

The code written for this article is openly available at (Github repository https://github.com/susanxschen/qldpc-fusion-lattices.).
