# Supplementary material for: Fusion-based implementation of qLDPC codes with quantum emitters
Source: npj Quantum Inf. 2026 Apr 13;12(1):90. doi: 10.1038/s41534-026-01233-y (PMC13265375; doi:10.1038/s41534-026-01233-y)
Supplement: Supplementary file 1 — Supplementary Information [file 41534_2026_1233_MOESM1_ESM.pdf]

Supplementary Information - Fusion-based implementation of qLDPC codes with quantum emitters

Susan X. Chen,[1,2,*] Matthias C. Löbl,[3,4] Ming Lai Chan,[3,4] Anders S. Sørensen,[3] and Stefano Paesani[2,†]

[1] *Quantum Engineering Centre for Doctoral Training, H. H. Wills Physics Laboratory and School of Electrical, Electronic, and Mechanical Engineering, University of Bristol, BS8 1FD, United Kingdom*

[2] *NNF Quantum Computing Programme, Niels Bohr Institute, University of Copenhagen, Blegdamsvej 17, DK-2100 Copenhagen Ø, Denmark*

[3] *Center for Hybrid Quantum Networks (Hy-Q), The Niels Bohr Institute, University of Copenhagen, DK-2100 Copenhagen Ø, Denmark*

[4] *Sparrow Quantum, Blegdamsvej 104A, DK-2100 Copenhagen Ø, Denmark*

I. DETAILS ON THE SPIN-BASED ARCHITECTURE WITH REPEAT-UNTIL-SUCCESS ENCODED FUSIONS

In practice, we consider the foliated lattice to be built layer by layer. To establish connectivity within layers, we use repeat-until-success (RUS) encoded fusions [1–3]. RUS fusions are encoded versions of physical fusions, where a physical fusion has the following success (s), failure (f) and erasure (l) probabilities:

$$P_s = P_f = \frac{1}{2}\eta^2 \quad \text{and} \quad P_l = 1 - \eta^2, \tag{S1}$$

Here $1-\eta$ is the photon loss probability given the total setup efficiency η. Due to the probabilistic nature of fusions, RUS is used to increase the success probability of a bond being created by allowing for multiple attempts in the event of physical failures.

We consider RUS fusions where a maximum of N repetitions can be performed. An RUS fusion has three different outcomes: (1) RUS fusion success, in which case a bond is established between the two spins, (2) RUS fusion failure, in which case there is no bond between the spins, (3) RUS fusion loss, in which case both spins need to be measured in the Z-basis, removing all their bonds. As a function of the photon loss probability the RUS fusion outcomes have the following probabilities [1]:

- The probability of RUS success, i.e., obtaining both outcomes, which corresponds to events in which $0 \leq i < N$ fusion failures occur before a successful fusion is given by: $P_N^{\bar{s}} = P_s \sum_{i=0}^{N-1} (P_f)^i$. In this case a connection between the two spins is established.
- The probability of RUS erasure, i.e., losing both outcomes, referring to events where the first i fusions failed and the subsequent fusions have photon loss is: $P_N^{\bar{l}} = P_l \sum_{i=0}^{N-1} (P_f)^i$. Here both spins need to be measured in the Z-basis.
- The probability of RUS failure, i.e., only obtaining one of the two outcomes, where all N repeats result in fusion failures is: $P_N^{\bar{f}} = (P_f)^N$. In this case, the connection between the two spins is not established.

We consider strategies for RUS sampling and scheduling, these can be understood with an approach from Ref. [4] to handle absent bonds in cluster state lattices.

Consider, for instance, the X-type lattice (the same reasoning applies to the Z-type lattice) - if a bond is missing, the two detector cells dependent on that bond (in both of the lattices) could be multiplied together to form a higher-weight *supercell* [5], thereby removing the cells' dependencies on the missing bond. One may, however, measure the qubit at either end of the missing bond in the Z-basis to prevent the merging of cells in one of the two lattices. We discard the measurement outcome and therefore this effectively is the same action as erasing the said qubit [4]. Figure S1(a) shows the presence of a missing bond (indicated in red) with respect to X-type detector cells, the choice to

* Corresponding author: susan.chen@nbi.ku.dk

† stefano.paesani@nbi.ku.dk

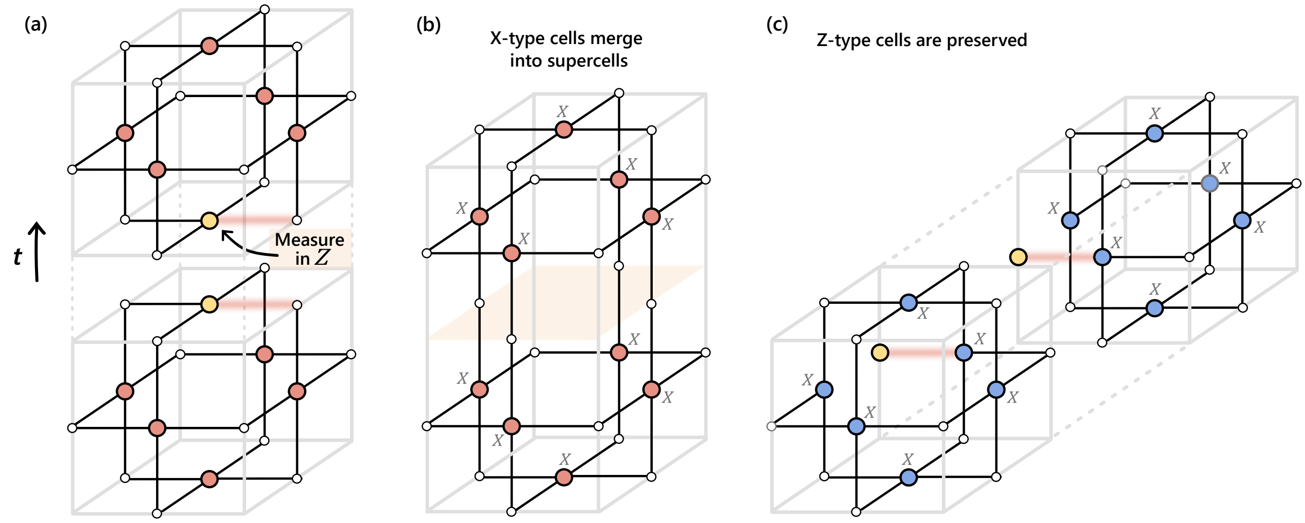

FIG. S1. Illustration of the missing bond strategy for topological cluster state lattices in Ref. [4] (a) Two neighbouring X-type detector cells of $X^{\otimes 6}$ on face qubits. If a bond is missing (marked in red), in this case we may choose to measure the (yellow) X-ancilla qubit in the Z-basis. The effect on the X-type lattice (b) is a supercell formed by merging the two cells, while on the Z-type lattice (c) is nothing and the cells stay intact.

measure the X-ancilla qubit (shown in yellow) merges the X-type cells as expected (Fig. S1(b)) while keeping the Z-type cells un-merged or *preserved* (Fig. S1(c)). Conversely, measuring the data qubit on the other end of the bond preserves the X-type cells and merges the Z-type cells.

We now consider the two strategies to handle imperfect RUS fusion. The first one, we refer to as *standard* RUS (and described in Ref [1]) and the second is the modified variant we devise and use in simulations.

1. All fusions within one layer are executed simultaneously. Upon fusion erasure, which dephases both spins adjacent to the fusion photons, we measure both spins in the Z-basis, which effectively erases them. This causes one merge in both the X and Z-type lattices. If we have a missing bond due to fusion failure, a Z-basis measurement is exclusively performed on the ancilla qubit. This strategy is equivalent to the one used in Ref [1].

2. All fusions are executed sequentially, where previous measurements inform subsequent fusions. A fusion may be omitted if either of the spins involved already needs to be measured in the Z-basis due to a previous fusion erasure or failure. Omitting these unnecessary fusions improves the loss tolerance. Our treatment of fusion erasure is the same as above, however for RUS fusion failure, we instead randomly choose the spin at either end of the missing bonds to be measured in Z.

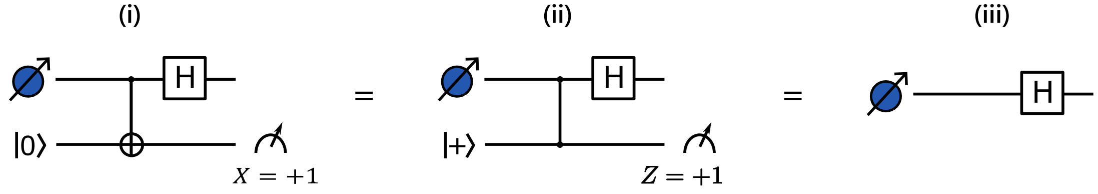

FIG. S2. Equivalent circuits to move spins (blue) from one layer of the lattice to the next. While ordinarily (i) a photon would be emitted and measured in X in the foliation picture, this is equivalent to (ii) a graph state with a dangling bond being measured in Z which is the same as (iii) omitting its generation.

We note that after all bonds in a given layer have been attempted by RUS fusions, H gates are applied to the spins to progress to the next layer. This can be understood by simultaneously executing the lattice construction and the teleportation of quantum information to the next layer by X-measurements. In Figure 1, a photon is first generated in place of the spin as a chain node. To teleport the encoded state forward in the lattice, this photon will subsequently be measured in the X-basis [6]. However, this circuit is equivalent to a circuit in which the photon is never generated (Fig. S2). This equivalence is important, as we therefore can omit the photon generation. These virtual qubits are thus never generated and so the only photons that can suffer loss are those involved in fusions.

In Figure S3, we show the reduced resource states after the X-basis projection of these qubits. The branched chains reduce down to branched chains of one less leaf per node, $(\eta_Q/2-1)$ leaves instead of $\eta_Q/2$, and the (η_A+1)-star graphs reduce down to η_A-star graphs, while acquiring single qubit H gates. The resulting lattice with virtual qubits omitted is shown in Figure S3 where some fusion bases (blue) are rotated by the H gates.

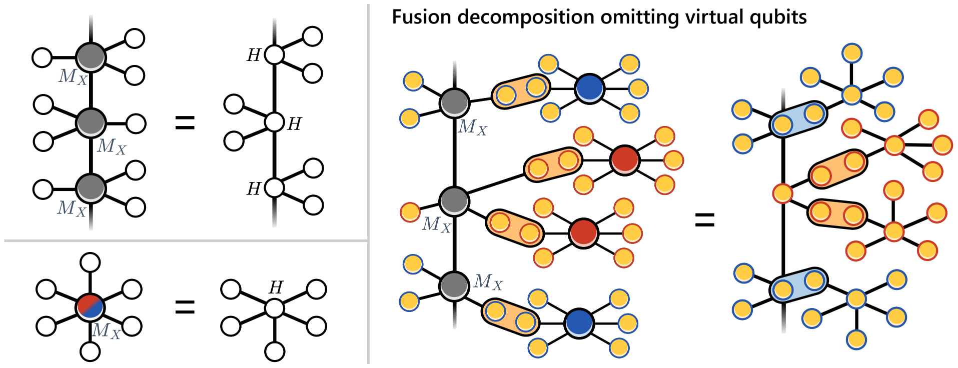

FIG. S3. Equivalent resource states having omitted virtual qubits via X measurement projection (M_X), here H indicates a Hadamard operation on the qubit. Fusion operations shown may also incur basis changes as a result, for instance, the blue ovals indicate the rotation of the original basis by a Hadamard gate.

The pseudo-thresholds and thresholds shown in Figure 3(b) are determined in the following way. Applying the modified RUS strategy, we numerically sample which encoded fusion outcomes are lost. Then we perform erasure decoding on the X-type lattice (10^5 Monte Carlo samples). Gaussian elimination is used to identify whether any of the k logical qubits are lost by checking if they have support on any of the missing fusion outcomes. We show the full data ($N=3$ to 10) obtained for Toric code and Bivariate Bicycle code lattices in Figures S4 and S5, respectively. Figure S4 also presents a comparison between the standard method and our modified strategy, highlighting the visible improvement from the latter.

II. MODIFIED FUSIONS IN REALISTIC SPIN-BASED ARCHITECTURES

In our architecture, we consider the situation that the fusion photons are two dangling bonds (leave nodes) attached to two spins. For the fusion-based construction of the foliated lattice shown in Fig. 1 of the main text we need a type of fusion that, up to local gates does the following: it makes (1) a CZ gate between the two spins upon success (add a graph bond), and (2) does two Z-basis measurements on the fusion photons upon failure (i.e. removes the nodes corresponding to the fusion photons). The first condition needs to be fulfilled to obtain the desired connectivity, and the second one ensures that the fusion can be repeated without any undesired effect on the graph state.

Labelling the two fusion photons as A, B, the standard fusion measures the parities X_AX_B, Z_AZ_B upon success and does the single-qubit measurements Z_A, Z_B upon failure [7]. In this case, fusion failure corresponds to removing the fusion qubits as desired, but the fusion success case does not result in the desired spin-spin connectivity. This issue is illustrated in Fig. A5(b) in Ref. [8]. In Fig. S6(a,b) we give two exemplary setups for dual-rail-encoded fusion qubits that work for our purposes. The first one in Fig. S6(a) is the standard fusion setup with an additional phase-shifter implementing an S-gate ($S = |0\rangle\langle 0| + i\,|1\rangle\langle 1|$) on the first qubit. Upon success, this setup measures the parities X_AY_B, Y_AX_B. It can easily be seen that this measurement has the desired effect up to measurement-dependent Pauli gates (not mentioned in the following) and two S-gates on both spins (see section D.1 in Ref. [8]). Upon fusion failure, the fusion measures Z_B, Z_B like the standard fusion and thus has the desired effect.

The second fusion setup is the standard fusion with an additional beam splitter and a phase-shifter implementing a Hadamard and an S-gate on the first qubit. This fusion measures the parities X_AY_B, Y_AZ_B upon success. Up to an S-gate on the spin connected to qubit B, this gives the desired CZ-gate between the spins (see Fig. A5(d) in Ref. [8] or Appendix D.2 in Ref. [9]). Upon fusion failure, Y_A, Z_B is measured which corresponds to removing the nodes A, B and applying an S-gate to the spin connected to qubit A (see e.g. Ref. [10]).

We finally remark that the additional S-gates commute with the gates applied to the spin as long as one only generates new photons entangled with the spin as dangling bonds (leaf nodes). This can be seen by considering the corresponding circuit from Fig.1(e) in the main text. This result is important from a practical point of view as it implies that the S-gates do not have to be actively removed between repeated fusions and not before an entire layer is finished.

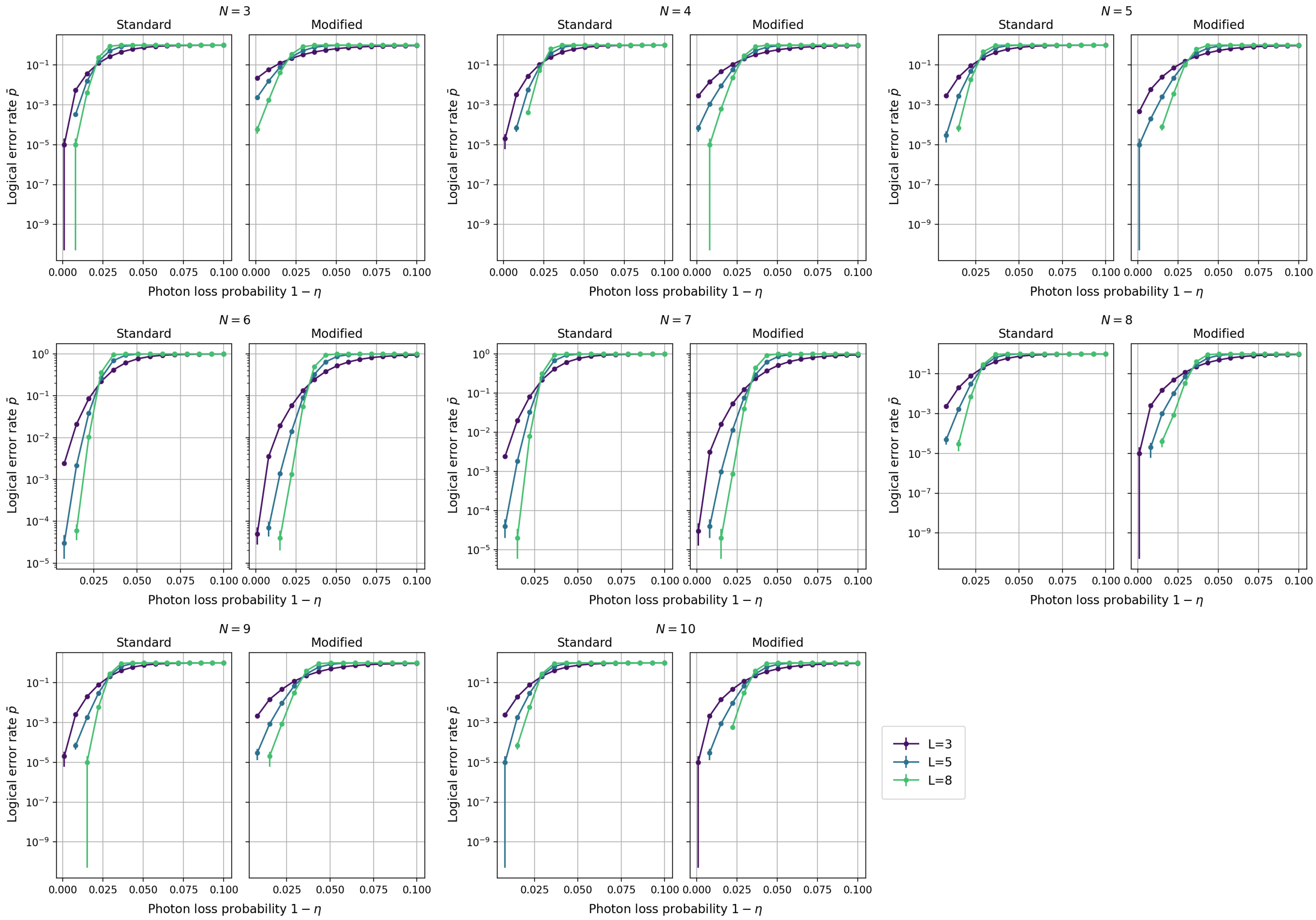

FIG. S4. Logical error rates $\bar{p}$ against photon loss probability $1-\eta$ for fusion lattices constructed from Toric codes of sizes $\{3, 5, 8\}$ under repeat-until-success with N, the number of maximum repeats, increasing from 3 to 10. In particular, a comparison is drawn between the standard strategy described in Ref. [1] and our updated modified strategy inspired by Ref. [4].

III. BIVARIATE BICYCLE QLDPC CODES

CSS codes are defined by binary matrices H_X and H_Z of size $(n-k) \times n$, which specify complete sets of X-type and Z-type stabiliser generators, respectively, with each row indicating the qubits on which a distinct stabiliser acts. The two types of stabilisers must be mutually commuting, in other words, satisfy the orthogonality relation:

$$H_X H_Z^T = 0 \pmod 2 \tag{S2}$$

Bivariate Bicycle qLDPC codes [11] are defined via the following parity check matrices

$$H_X = [A|B] \quad \text{and} \quad H_Z = [B^T|A^T], \tag{S3}$$

where A and B are sums of matrices $A = \sum_i^3 A_i$ and $B = \sum_i^3 B_i$, with A_i and B_i being powers of

$$x = S_l \otimes I_m \quad \text{and} \quad y = I_l \otimes S_m, \tag{S4}$$

with S_l being the cyclic shift matrix of size $l \times l$ and I_m being the $m \times m$ identity matrix. A code defined as such will have a degree-6 Tanner graph, in contrast to 4 for the surface code. We select a number of small Bivariate Bicycle qLDPC code examples to construct and test our lattice constructions. These are shown in table S1. One can prove, due to certain group properties, that these codes can be presented in a *Toric layout*, meaning all vertices of

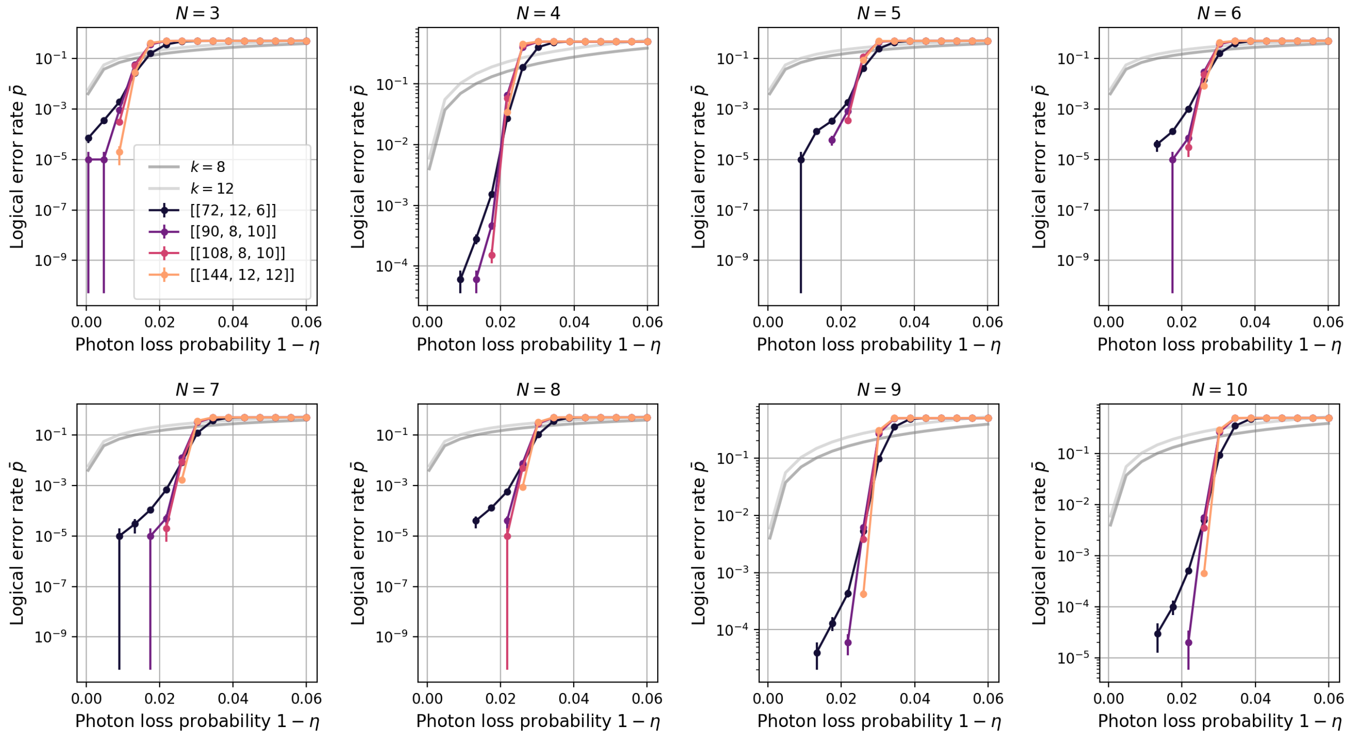

FIG. S5. Logical error rates $\bar{p}$ against photon loss probability $1-\eta$ for fusion lattices constructed from small examples of Bivariate Bicycle qLDPC codes under repeat-until-success with N, the number of maximum repeats, increasing from 3 to 10. As previously, the grey curves are the break-even equations for $k = 8, 12$.

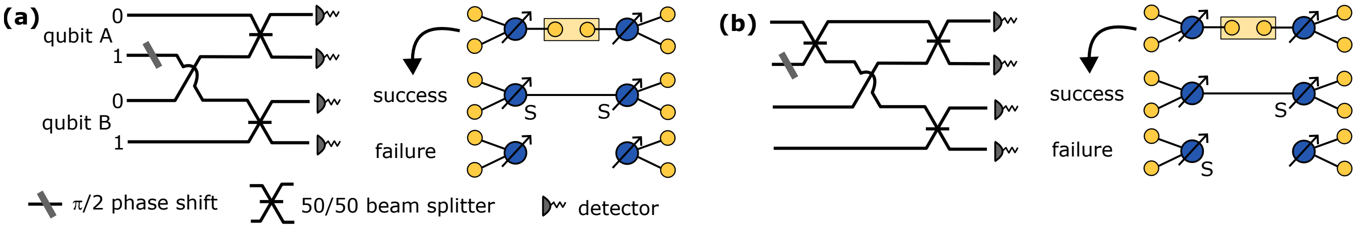

FIG. S6. Two fusion setups for dual-rail encoded fusions. Up to local S-gates, both fusions have the desirable property to realize a connection between the two spins upon fusions success, while only removing the fusion photons upon fusion failure.

the Tanner graph can be placed on a planar grid with periodic boundary conditions, like the Toric code [11]. In this layout, each vertex has horizontal and vertical edges connecting to its nearest neighbour vertices, forming a subset local embedding of the code, while the remaining two edges at each vertex correspond to non-local connections. A section of the Tanner graph of the smallest considered code $[[72, 12, 6]]$ is presented in its Toric layout in Figure 1(b) of the main text.

$[n, k, d]$	l, m	A	B	Error pseudo-threshold (%)	Erasure pseudo-threshold (%)
$[[72, 12, 6]]$	$6, 6$	$x^3 + y + y^2$	$y^3 + x + x^2$	0.147	8.07
$[[90, 8, 10]]$	$15, 3$	$x^9 + y + y^2$	$1 + x^2 + x^7$	0.158	8.41
$[[108, 8, 10]]$	$9, 6$	$x^3 + y + y^2$	$y^3 + x + x^2$	0.176	8.53
$[[144, 12, 12]]$	$12, 6$	$x^3 + y + y^2$	$y^3 + x + x^2$	0.181	8.70

TABLE S1. Examples of small Bivariate Bicycle qLDPC codes from Ref. [11] with their defining parameters and pseudo-thresholds of the corresponding fusion lattices.

[1] G. de Gliniasty, P. Hilaire, P.-E. a. W. S. C. Emeriau, A. Salavrakos, and M. Shane, A spin-optical quantum computing architecture, Quantum **8**, 1423 (2024).
[2] M. L. Chan, T. J. Bell, L. A. Pettersson, S. X. Chen, P. Yard, A. S. Sørensen, and S. Paesani, Tailoring fusion-based photonic quantum computing schemes to quantum emitters, PRX Quantum **6**, 020304 (2025).
[3] M. C. Löbl, S. Paesani, and A. S. Sørensen, Efficient percolation simulations for lossy photonic fusion networks, Phys. Rev. Res. **6**, 033273 (2024).
[4] J. M. Auger, H. Anwar, M. Gimeno-Segovia, T. M. Stace, and D. E. Browne, Fault-tolerant quantum computation with nondeterministic entangling gates, Phys. Rev. A **97**, 030301 (2018).
[5] S. D. Barrett and T. M. Stace, Fault tolerant quantum computation with very high threshold for loss errors, Phys. Rev. Lett. **105**, 200502 (2010).
[6] R. Raussendorf, D. E. Browne, and H. J. Briegel, Measurement-based quantum computation on cluster states, Phys. Rev. A **68**, 022312 (2003).
[7] S. Bartolucci, P. Birchall, H. Bombin, H. Cable, C. Dawson, M. Gimeno-Segovia, E. Johnston, K. Kieling, N. Nickerson, M. Pant, *et al.*, Fusion-based quantum computation, Nat. Commun. **14**, 912 (2023).
[8] M. C. Löbl, S. Paesani, and A. S. Sørensen, Loss-tolerant architecture for quantum computing with quantum emitters, Quantum **8**, 1302 (2024).
[9] M. C. Löbl, L. A. Pettersson, S. Paesani, and A. S. Sørensen, Transforming graph states via Bell state measurements, Quantum **9**, 1795 (2025).
[10] M. Hein, W. Dür, J. Eisert, R. Raussendorf, M. Nest, and H.-J. Briegel, Entanglement in graph states and its applications, arXiv quant-ph/0602096 (2006).
[11] S. Bravyi, A. W. Cross, J. M. Gambetta, D. Maslov, P. Rall, and T. J. Yoder, High-threshold and low-overhead fault-tolerant quantum memory, Nature **627**, 778–782 (2024).
